# Contextualization of psychological treatments for government health systems in low-resource settings: group interpersonal psychotherapy for caregivers of children with nodding syndrome in Uganda

**DOI:** 10.1186/s13012-018-0785-y

**Published:** 2018-06-28

**Authors:** Byamah B. Mutamba, Brandon A. Kohrt, James Okello, Janet Nakigudde, Bernard Opar, Seggane Musisi, William Bazeyo, Joop de Jong

**Affiliations:** 1 Butabika National Mental Hospital, Kampala, Uganda; 2 Amsterdam Institute of Social Science Research, Faculty of Social and Behavioural Sciences, University of Amsterdam, Amsterdam, The Netherlands; 3 Department of Psychiatry, George Washington University, Washington, DC USA; 4 Department of Psychiatry, Gulu University, Gulu, Uganda; 5 Department of Psychiatry, College of Health Sciences, Makerere University, Kampala, Uganda; 6 Ministry of Health Headquarters, Kampala, Uganda; 7 Makerere University School of Public Health, Makerere University, Kampala, Uganda

**Keywords:** Adaptation, Contextualization, Lay community health workers, Public health system, Implementation, Caregivers, Nodding syndrome, Group interpersonal psychotherapy

## Abstract

**Background:**

Evidence for the effectiveness of psychological treatments in low- and middle-income countries is increasing. However, there is a lack of systematic approaches to guide implementation in government health systems. The objective of this study was to address this gap by employing the Replicating Effective Programs (REP) framework to guide contextualization of a psychological treatment in the Uganda public health system for caregivers of children affected by nodding syndrome, a neuropsychiatric disorder endemic to Sub-Saharan Africa associated with high morbidity and disability.

**Methods:**

To contextualize a psychological treatment, we followed the four components of the REP framework: pre-conditions, pre-implementation, implementation, and maintenance and evolution. A three-step process involved reviewing health services available for nodding syndrome-affected families and current evidence for psychological treatments, qualitative formative research, and analysis and documentation of implementation activities. Stakeholders included members of affected communities, health care workers, therapists, local government leaders, and Ministry of Health officials. Detailed written, audio, and video documentation of the implementation activities was used for content analysis.

**Results:**

During the pre-condition component of REP, we selected group interpersonal therapy (IPT-G) because of its feasibility, acceptability, effectiveness in the local setting, and availability of locally developed training materials. During the pre-implementation component, we adapted the training, logistics, and technical assistance strategies in conjunction with government and stakeholder working groups. Adaptations included content modification based on qualitative research with caregivers of children with nodding syndrome. During the implementation component, training was shortened for feasibility with government health workers. Peer-to-peer supervision was selected as a sustainable quality assurance method. IPT-G delivered by community health workers was evaluated for fidelity, patient outcomes, and other process-level variables. More than 90% of beneficiaries completed the treatment program, which was effective in reducing caregiver and child mental health problems. With the Ministry of Health, we conducted preparatory activities for the maintenance and evolution component for scale-up throughout the country.

**Conclusions:**

The REP framework provides a systematic approach to guide contextualization of psychological treatments for delivery in low-resource public health systems. Specific recommendations are provided for REP’s application in global mental health.

**Trial registration:**

ISRCTN11382067; 08/06/2016; retrospectively registered

**Electronic supplementary material:**

The online version of this article (10.1186/s13012-018-0785-y) contains supplementary material, which is available to authorized users.

## Background

Successful scaling-up of evidence-based health interventions requires “contextualization,” which refers to adapting and integrating interventions into the “prevailing physical, socioeconomic, cultural, health systems, stakeholder, and institutional culture[s]” that “contribute to and affect the planning, implementation, monitoring, and outcomes of interventions” [1]. Although there is increasing attention to the need for systematic approaches to cultural adaptation of evidence-based treatments (EBTs) [2, 3], the process of contextualization has received limited attention. Whereas cultural adaptation typically focuses on content modification, such as including local explanatory models for the target beneficiary population, EBTs also require modification based on health system structures and policies, professional background of health care providers, and availability of other psychosocial resources in the health system and community [1]. Although cultures vary widely around the world, there are common contextualization challenges faced by government health systems in low- and middle-income countries (LMIC) and in other low-resource settings. There are increasing attempts to integrate EBTs into government settings, with limited publications to date on how the process is done and a lack of specific implementation strategies to guide it [4]. Therefore, there is a global need to develop common guidance principles for contextualization of EBTs.

One domain in which contextualization for low-resource settings is especially needed is the delivery of psychological treatments (PTs), which are rarely available in LMIC [5, 6]. Psychological treatments delivered by non-specialists in LMIC can achieve effectiveness comparable to delivery by specialists in high-resource settings [7]. This process, known as task-shifting or task-sharing, has been applied across LMIC [8, 9]. In Sub-Saharan Africa, group interpersonal therapy (IPT-G) delivered by lay community health workers (LCHWs) is a feasible and effective intervention for treatment of depression [10–12]. In Uganda, IPT-G underwent cultural adaptation [13, 14] and was effective in community settings when delivered by an international non-governmental organization [10, 15–18]. However, IPT-G and other PTs have not been routinely integrated in Uganda and other national health systems. Because government health systems provide the majority of care in LMIC, systematic contextualization processes for EBTs are crucial. We aimed to use an implementation framework to guide the process so that we could learn if and how this methodology would be beneficial across health systems in LMIC.

Literature on HIV/AIDS interventions can be a guide for systematic contextualization and scaling-up of other types of interventions, including mental health care, within government health systems. With proliferation of effective HIV interventions, the US Centers for Disease Control and Prevention developed the Replicating Effective Programs (REP) framework [19, 20]. The hallmark of REP is balancing fidelity to the core intervention components while promoting flexibility to maximize the diversity of settings for implementation [20]. REP is divided into four phases: (i) pre-conditions, (ii) pre-implementation, (iii), implementation, and (iv) maintenance and evolution [20, 21]. Randomized controlled trials employing REP have demonstrated greater uptake and fidelity of interventions [20, 22]. In the context of health care interventions, the pre-condition phase consists of identifying and packaging of the intervention for training and assessment; pre-implementation consists of customization, training, and establishing technical assistance services; implementation consists of delivery and evaluation; and maintenance and evolution requires models for financial sustainability, nationwide dissemination, and iterative re-customization [20].

Within the field of implementation science frameworks, REP is considered a *prescriptive* framework, which contrasts with other frameworks that are more descriptive, explanatory, or predictive in nature [23]. We needed a prescriptive framework in order to guide our contextualization process to optimize implementation in a government setting. Moreover, among 49 implementation frameworks reviewed, REP was one of only two frameworks that included a range of items within the strategies for implementation domain [23].

The REP prescriptive framework was well suited to address two contemporaneous endeavors in Uganda. First, the Uganda government recently developed national health guidelines for management of nodding syndrome (NS) [24], a neuropsychiatric disorder endemic to Sub-Saharan Africa associated with high levels of morbidity and disability [24–26]. The national management guidelines target the affected children using a multidisciplinary treatment approach; however, no treatments specific to the caregivers were established [24]. A PT is needed to address distress experienced by caregivers of children with NS. Second, the World Health Organization (WHO) and Uganda Ministry of Health are collaborating for adaptation and contextualization of the mental health Gap Action Programme (mhGAP) [27], which includes plans to pilot a PT within government health services for subsequent scaling-up throughout the country. The WHO mhGAP intervention guide is a framework for delivery of mental health services within primary care settings, designed with intention for delivery in government health services [27]. Contextualization of IPT-G into government health systems addresses both the need for NS services and PTs available for primary care.

Psychological treatments for caregivers of children with NS are vital because of the high burden of psychological distress associated with caring for these children and because of the high levels of community stigma experienced by caregivers and their children [28]. NS is described as a progressive epileptic encephalopathy which afflicts persons between the ages of 3 and 18 years [29]. In northern Uganda, the clinical presentation is heterogeneous typified by head nodding episodes and other mental and physical health problems [24]. Video documentation of these episodes is available (https://youtu.be/TQk2ZMy9O1k). NS can be complicated by co-morbidity with mental health problems and idioms of distress in the affected child and his/her caregiver [24]. Debilitating physical symptoms associated with negative attitudes and social stigma may contribute to depression and anxiety symptoms in those affected and their caregivers [24, 28]. This may in turn exacerbate NS symptoms because of increased stress in the family and socio-cultural environment [24, 30].

Our objective, therefore, was to contextualize and implement a PT for delivery within the government health system in Uganda, with the intended primary outcome of reducing common mental health problems among caregivers and their children affected by NS. In this process, we also sought to explore REP as a systematic framework for contextualizing PTs in other low-resource settings.

## Methods

Activities were conducted to address the four components of the REP framework: pre-conditions, pre-implementation, implementation, and maintenance and evolution (Table 1). In addition to contextualization, standard approaches for cultural adaptation were employed for the specific population needs related to NS [3]. Research was conducted from April 2013 to August 2014.

**Table 1:** Replicating Effective Programs (REP) framework applied to contextualization of a psychological treatment, group interpersonal therapy (IPT-G), within the Uganda public health system

REP component	Component elements used	Methods: Uganda activities
Pre-conditions	1.1 Identify need for intervention	1.1 Review of government NS program response highlighted mental health needs of caregivers of children with NS
	1.2 Identify intervention for local setting	1.2 Desk review of pre-existing literature on EBTs in Uganda led to selection of IPT-G based on prior effectiveness demonstrated in NGO settings
	1.3 Package intervention for training and assessment	1.3 Review of literature and consultations with IPT therapists revealed IPT-G materials previously developed with illustrations and Luo language adaptation
Pre-implementation	2.1 Orientation to core elements	2.1 Stakeholder meeting with government administrative health officials and IPT-G experts refined and agreed on core elements
	2.2 Customize delivery	2.2 Adaptation of package based on formative research with families affected by NS and contextualization to government health system delivery
	2.3 Identification of barriers	2.3 Coordination with existing NS health service delivery
	2.4 Staff training needs	2.4 Identification of government health staff to be trained in IPT-G delivery
	2.5 Technical assistance needs	2.5 Supervision to be carried out by IPT-G experts in Uganda and study team
Implementation	3.1 Ongoing community partnership	3.1 Coordination with government health system and local community during delivery of IPT-G
	3.2 Training and technical assistance	3.2 Training of village health team members to implement IPT-G. Training of government health workers to be field supervisors. Establish weekly supervision meetings between village health teams and their health worker supervisors facilitated by IPT-G experts
	3.3 Process evaluation	3.3 Collection of video, audio, and written documentation from trainings, IPT-G weekly sessions, supervision, and coordination meetings; controlled before and after study with evaluation of caregiver and child outcomes
	3.4 Feedback and refinement of intervention packageand training	3.4 Post-intervention feedback from government health system workers, village health team members, IPT-G recipients, and community members
Maintenance and evolution	4.1 Organizational and financial changes to sustain intervention	4.1 Costs of delivering IPT-G for NS affected families estimated
	4.2 Prepare package for national dissemination	4.2 Intervention manualized, materials and implementation plan modified for integration in national guidelines for NS health services
	4.3 Re-customize delivery as need arises	4.3 Plan to conduct validation study to incorporate livelihoods scheme, develop quality assurance and customization guidelines

*Abbreviations*: *EBT* evidence-based treatment, *HWs* health workers, *IPT-G* group interpersonal therapy, *NGO* non-governmental organization, *NS* nodding syndrome, *REP* Replicating Effective Programs

### Component 1. Pre-conditions

#### Document reviews

At the initiation of the study, the team reviewed existing government health service guidelines for NS and psychosocial, psychological, and mental health services. We conducted a review of literature on existing evidence-based PTs by non-specialist health workers that had previously been conducted in Uganda [10, 11]. Limiting searches to Uganda, we searched using the keywords: “psychological treatment,” “psychological intervention,” “psychotherapy,” or “psychological therapy.” We searched Ovid MEDLINE, PsycINFO, PubMed (including in-process citations, ahead of print), and Web of Science.

### Component 2. Pre-implementation

#### District consultation meetings and implementation stakeholder workshops

**Fig. 1:**
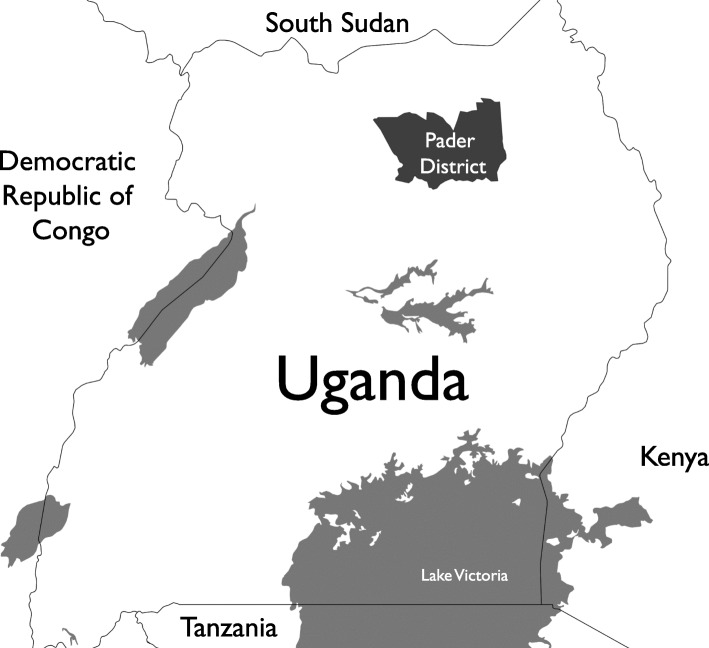
Uganda government health system structure

**Fig. 2:**
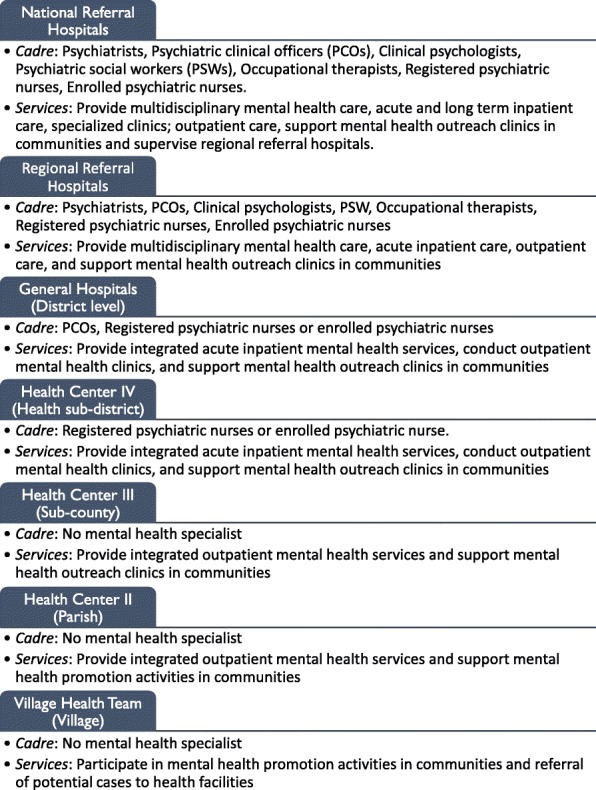
Map of Uganda showing Pader district

The health system in Uganda is organized into seven levels, and mental health is included in the Minimum Health Care Package provided at all levels of care (Fig. 1) [31]. The study was conducted in Pader district which had more than 50% of reported NS cases in northern Uganda [29] (Fig. 2). The district is subdivided into 12 sub-counties, each served by at least one level II and III health facility. The two study sub-counties each had one health facility designated as a NS treatment center. Atanga sub-county, the intervention site, had a population of 34,103 in 2014 at the time of the program [32].

The principal investigator and principal medical officer (PMO) in charge of NS response at the MOH held face-to-face consultative meetings with 11 stakeholders including district administrators and leaders, health workers, and representatives of NGOs. The goals of the meetings were to develop a partnership with the district leadership, establish administrative consent, and produce a joint agreement on the specific study sites, taking into account existing NGO programs. A 2-day stakeholder contextualization workshop was held with study investigators, representatives from the mental health division of the MOH and PT specialists who had participated in prior IPT-G randomized controlled trials (RCTs) in Uganda. The objective of the meeting was to inform the structure and content of the PT within the public health system, which would then inform the planned pilot of WHO mhGAP in Uganda. The meeting involved three activities: (a) presentations to stakeholders about the proposed study design, the national response to NS, the Uganda public health system, and the experience of World Vision Uganda (an NGO which conducted community-based delivery of IPT-G); (b) group work to make suggestions about possible adaptations to the structure and content of the intervention; and (c) plenary discussions to agree on how the adapted PT structure, content, and process could be implemented.

#### Formative study with beneficiaries

A qualitative study of caregiver burden for children with NS including focus group discussions (FGD) and in-depth interviews (IDI) was conducted. Results from this study informed the training program, supervision, structure, and content of the intervention [28].

### Component 3. Implementation

#### Training of village health team members (VHTs) and health workers

The IPT-G therapists conducted a 7-day training in the local language, Luo, for VHTs from the intervention site. A mixture of didactic lectures and role plays for the first 5 days was used, followed by role plays of the treatment sessions for 2 days. Broad thematic learning areas included definition and presentation of NS, caregiver experiences as described in the formative study, symptoms of mental illness with a focus on depression, working in groups, principles and practice of counseling, IPT-G elements, documentation, and supervision. A 3-day refresher training on the medical management of children with NS was given to all participants in accordance with government guidelines [24]. After the seventh treatment session, a 2-day refresher training was held for VHTs from the intervention site using a variety of teaching methods including lectures, experiential teachings, storytelling, group exercises, and role plays. A rapporteur compiled daily session and activity reports to inform the training evaluations each day. Thirteen VHTs and two health workers from the supervising health facility attended the initial training. Ten VHTs and the two health workers attended the refresher training.

#### Recruitment of intervention participants

With support from the national coordinator for NS, we sought and received permission from the district administration to access demographic records of caregivers of children with NS. With the registry, VHTs identified caregivers and informed them about the study. Caregivers providing consent were administered the pre-intervention assessment form (Additional file 1). This form recorded the number of depressive symptoms based on the Patient Health Questionnaire (PHQ-9), level of functioning, problem areas, and therapy goals. Therapists together with the VHTs and health workers reviewed the pre-group assessment forms to determine which caregiver was eligible to join a therapy group. A PHQ-9 score ≥ 9 was considered indicative of depression and therefore eligible to participate. The PHQ-9 has demonstrated high specificity and sensitivity in screening for depressive symptoms in Uganda [33].

#### Delivery of psychological treatment within the government health system

The intervention was conducted in seven villages of Atanga sub-county, involving 12 weekly treatment sessions with caregivers of children with NS. VHTs facilitated the IPT-G sessions, some of which were attended by the primary health workers who monitored adherence to the IPT-G process using a supervision checklist. Both the VHTs and the health workers received technical and support supervision from the IPT-G therapists and a clinical psychologist at weekly group supervision meetings. Weekly session and field supervision reports were prepared by VHTs, health workers, and a supervising clinical psychologist. IDIs were conducted with a sample of participating caregivers to document experiences of the intervention. Video documentation of sessions was done to guide supervision and increase treatment fidelity. The treatment-as-usual (TAU) control arm comprised care to caregivers of children with NS as recommended by the government guidelines [34].

#### Process and outcome evaluation

We conducted post-intervention assessment exercises to explore caregivers’ experiences of the group intervention from the perspective of the VHTs and other members of the intervention team. Two weeks after completion of the group intervention, VHTs conducted a post-group assessment exercise among the 69 caregivers who completed the 12 group sessions. A post-group test instrument (Additional file 1) was used to assess depressive symptoms and functioning. Therapists, VHTs, and health workers reviewed the post-group assessment forms to determine which caregiver had reduction in their symptoms and if they achieved their therapy goal.

#### Feedback and dissemination meetings

After individual caregiver post-group assessments, a debriefing meeting was held for VHTs. In addition, a half-day meeting was held 6 months after the group intervention, between investigators, research assistants, project administrators, and the implementers of the intervention. Study investigators reviewed the meeting minutes to identify lessons learned, challenges, and opportunities for scale-up. For dissemination, a 1-day community meeting was held at the study site, 8 months after completion of the intervention. Presentations from the national coordinator of the NS response, district officials, VHTs, and study team members were supplemented by testimonies from participating caregivers.

### Component 4. Maintenance and evolution

#### Scale-up activities

We conducted preparatory activities to inform scale-up of the intervention. Following dissemination of results at the study site, the study team developed a VHT training guide (available from first author) including adaptations made during implementation. We estimated costs of implementing components of the intervention package.

### Analysis

A process analysis was done during and at the end of each implementation phase to inform the next, and a qualitative analysis was conducted to complement the process evaluation**.** Qualitative analysis was done using stakeholder meeting notes, activity and supervision reports, process notes from IPT-G sessions, and audio and video recordings from meetings and intervention sessions. Audio and video files were transcribed into English by Luo-speaking research assistants. For each data source, summary notes were compiled and typed into a word document to highlight action points, challenges, and key messages. The transcripts and summary notes were then analyzed with NVivo software [35] using a framework analysis approach with predefined themes: cultural adaptations, contextual adaptations, acceptability, feasibility, fidelity to IPT-G structure and process, barriers to implementation, mitigation strategies for barriers, and recommendations for implementation. The choice to use predefined themes rather than deductive coding is based on qualitative approaches in which previous literature and theory suggest that particular themes are commonly associated with the key research question [36]. In the case of this study, there were predefined themes selected from common domains in implementation research [37] and domains relevant to adaptation of global mental health interventions [5]. The first author read through all transcripts and developed a codebook based on the themes above including criteria for coding and examples. Two independent coders then each reviewed all transcripts. They practiced coding transcripts until they reached an inter-rater reliability (*kappa*) of 0.79. Quantitative pre-post-intervention comparison results have been published separately [34].

## Results

### Pre-conditions

#### Document reviews

The literature review identified 17 publications in Ovid MEDLINE, 31 in PsycINFO, 18 in PubMed, and 14 in Web of Science as of December 31, 2012. After removal of duplicates, there were 34 publications on PTs in Uganda. Nine publications presented results from four RCTs (see PRISMA figure in Additional file 2). The trials include two types of PTs in Uganda: group interpersonal psychotherapy (IPT-G) [10, 11] and Narrative Exposure Therapy (NET) [38, 39]. Both IPT-G and NET had demonstrated efficacy in treatment of mental disorders in adult and adolescent populations. Both trials for treatment of war-affected adolescents were conducted in northern Uganda, a setting similar to that of our study. No prior studies had evaluated a PT in a government health setting.

When determining which of the PTs to select, we found that IPT-G had published literature on adaptation and training [13, 14] which would facilitate development of intervention materials. Though NET trials utilized a cadre similar to VHTs used in our study, NET requires a strictly manualized process for delivery [38]. Because of this, researchers developed another treatment; trauma counseling (TC) to simulate how NET might be used out of a trial setting in a regular care system and that allowed counselors to be flexible in their treatment delivery [38]. However, this was not standardized and would pose challenges in ensuring fidelity to treatment by the lay counselors. In addition, locally adapted materials for low literacy populations in Luo existed for IPT-G that necessitated minimal adaptations for use by VHTs. Training in NET lasts 6 weeks for lay counselors [38] unlike training for IPT-G which lasted 2 weeks at most [13], making it a more feasible option. Of note, group treatments were more culturally acceptable [28] and delivery of IPT-G weekly sessions seemed to be more logistically feasible than the individual NET treatments which required more human and financial resources [28]. Our primary study outcome was depression, and the evidence of treatment effectiveness for depression is stronger for IPT-G in Uganda compared to NET [10, 11, 38, 39]. In comparison to individual treatments, group PTs are thought to have other potential benefits including increased social support, acceptance, hope, and belonging among distressed individuals [40, 41]. Furthermore, IPT is a WHO mhGAP recommended treatment unlike NET and with sustainability in mind, using IPT-G would inform scale-up of psychosocial interventions in line with the MOH strategic plan. Therefore, IPT-G was selected as a potential treatment for caregivers of children with NS.

### Pre-implementation

#### Consultation meetings

A number of adaptations to the study design were suggested during the stakeholder adaptation meeting (Table 2).These included shorter periods of training for VHTs compared to that for IPT therapists in previous IPT-G interventions in Uganda in which training was for 2 weeks. Unlike NGO workers who were paid to deliver IPT-G, government VHTs are volunteers and can only afford to be away from their homes and/or livelihoods for a few days. Uganda national guidelines recommend 2 days per training for VHTs. However, following advice from MOH, a 7-day training program for VHTs was acceptable. Another modification was separating health workers from VHTs for training. This would maintain the expected Ugandan healthcare hierarchical structure.

**Table 2:** Summary of pre-implementation phase adaptations

Method	Findings
Consultation meetings and stakeholder adaptation workshop	• Government health officials emphasized the lack of mental health support for caregivers of children with nodding syndrome, and they agreed with the proposed study design using interpersonal therapy for groups (IPT-G).
	• Health officials and other government leaders recommended study sites.
	• A group size of 8–12 members was recommended, with one group per village based on health records of households with children living with NS in proposed study sites.
	• It was agreed that members of village health teams (VHTs) would deliver IPT-G to caregivers of children affected by NS.
	• Stakeholders in the adaptation workshop suggested that 12-session IPT-G would be feasible. Mixed gender groups were considered acceptable by the community because both male and female caregivers face similar challenges with children living with NS.
	• Health officials set a limit of fewer than 14 days for training of VHTs to minimize disruption of other work and personal activities. They recommended a 5-day workshop type of training and 2 days of role plays to practice implementation of IPT-G.
	• To maintain the hierarchical divide between primary health workers and VHTs, health workers would be oriented to IPT concepts, design, and supervision activities in separate sessions conducted in parallel with VHT training. Health workers would attend some VHT training sessions to appreciate the practicalities of IPT-G intervention.
	• Previously trained Ugandan IPT-G therapists, working together with the study clinical psychologist would supervise both the health workers and VHTs for technical assistance.
	• IPT groups would be co-facilitated by 2 VHTs to ensure continuity of the group sessions in the event that one of the VHTs was absent.
Qualitative study with caregiver beneficiaries	• Three themes linked to caring for a child with nodding syndrome were identified as targets for IPT-G: (1) agony resulting from community stigma and fear; (2) cognitive, emotional, and behavioral disturbances; and (3) physical and financial constraints.

Modifications were made to tools used in previous IPT-G studies. To assess caregiver depression, stakeholders chose to use a Luo version of the PHQ-9 questionnaire rather than the Acholi Psychosocial Assessment Instrument (APAI), which was used in the prior studies [11]. The PHQ-9 has been used in primary care settings within Uganda [42] and in contrast to nine items for the PHQ, the APAI is a greater burden for participants because it has 60 items. In addition, the APAI which was developed for adolescents emotional and behavioral problems, *malwor* and *gin lugero* [43], was not applicable to younger children and adult caregivers in our program. The measure of caregiver functioning (Additional file 1) was similar to that used in previous studies [10, 11, 44]. Stakeholders agreed with the study proposal to have VHTs deliver the intervention and health workers as supervisors of VHTs. IPT-G therapists would be overall supervisors of the intervention delivery.

An additional modification was that therapy groups would be co-facilitated by two VHTs of different gender and educational levels rather than only one VHT as we originally proposed. Dual facilitation would assure continuity of the group sessions in the event that one of the VHTs was absent. Government staff, following observations by the IPT-G therapists, supported the decision to shorten the original 16-session IPT-G conducted in Uganda [10] to a 12-session structure (two initial sessions, eight middle sessions, two termination sessions) which would increase the likelihood of delivery when scaled-up in government settings.

#### Formative study with beneficiaries

Family challenges associated with caring for children affected by NS, particularly those related to caregiver mental health, were identified [28]. Key contextualization issues included acceptance of facilitators of either gender (vs. gender-specific matching of facilitators and groups in earlier trials) or a focus on a specific problem (nodding syndrome vs. focusing on a range of psychological stressors).

### Implementation

#### Training of VHTs and health facility workers

The training of VHTs was conducted in 7 days. Training was participatory with VHTs contributing to the modification of materials, such as translation of concepts to less technical, culturally sensitive language to be used in intervention delivery (Table 3**)**. Common local terms representing symptoms of depression, e.g., *cwer cwinyi* (feeling sad or hopeless), were suggested and agreed upon by VHTs. This exercise also informed further translation of the intervention tools including in-session and pre/post assessment forms. Orientation of health workers to IPT-G supervision was done by one of the IPT-G therapists, in a separate session during the course of the VHT training.

**Table 3:** Summary of implementation phase adaptations

Method	Findings
Primary training of VHTs and government health workers	• Training curriculum was modified for a 7-day curriculum with content derived from WHO mhGAP-IG materials for community health workers, the IPT-G manual previously used in Uganda [13], recommendations from the stakeholder consultative meeting, and objectives of the IPT-G for NS study.
	• Core components of intervention were considered to be: identification of depressive symptoms and their effects on each group member’s life, articulation of the link between interpersonal problems and symptoms of depression, exploration of triggers of depression, classification of triggers into one IPT problem area (grief, role disputes, role transition, or interpersonal deficits), and collaborative establishment of practical treatment goals with caregivers.
	• For supervision training, previously trained Ugandan IPT therapists conducted an orientation of health workers to IPT-G processes in a half-day session held in parallel to VHT training.
Recruitment within government health systems	• VHTs were able to identify 86 caregivers to participate in the pre-group exercise and 73 fulfilled criteria (PHQ score ≥ 9) for inclusion in the therapy groups. However, participants with low PHQ scores were allowed to participate after they requested to participate to learn IPT skills for managing stress associated with caregiving for children with NS. Two additional caregivers self-presented after the intervention had started and were included as well.
Delivery of IPT-G in government health systems	*Initiation phase*
	• Sessions 1 and 2: common presenting problems were life changes associated with caring for children with NS, experiencing domestic violence, living with HIV/AIDS, and poverty. Caregivers disclosed suicide ideation, and additional training and supervision were provided in making referrals to health facilities and follow-up of caregivers with suicidal behavior.
	*Middle phase*
	• Sessions 3 and 4: caregivers displayed increasing openness to share problems and explore solutions.
	• Session 5: as caregivers were expected to provide more concrete weekly and long-term goals, they expressed difficulty in articulating these targets. VHTs reported difficulties completing group session forms for this phase of treatment. To address the documentation challenge, peer supervision by VHTs who were more literate was used to support documentation for less educated VHTs and for other VHTs with difficulties understanding documentation.
	• Session 6: VHTs noted that the caregivers not showing symptomatic improvement were those experiencing ongoing domestic violence. In response, supervision sessions were dedicated to exploring and supporting caregivers experiencing domestic violence.
	• Session 7: VHTs displayed increasing competence in basic IPT concepts and group facilitation techniques. VHTs reported difficulty engaging caregivers who were reporting symptom resolution, e.g., these caregivers did not see the merit in returning for further sessions. IPT-G therapists made group session visits to observe challenges and tailor content of refresher trainings.
	• Sessions 8, 9, and 10: caregivers testified about the positive effects of the IPT-G on their lives.
	*Termination phase*
	• Sessions 11 and 12: the majority of caregivers expressed positive future plans. Some of the groups decided to continue as self-help support and income generating groups (*Bol Cup*). VHTs continue to voluntarily engage with caregivers in the community and encourage their self-care.
	*Supervision and booster/refresher training*
	• VHT implementation challenges were used to inform supervision sessions and a booster/refresher training was conducted after session 7, with a focus on preparing for subsequent phases and understanding the need and implementation of the termination phase of IPT-G. Supervision focused on adherence to IPT-G structure and addressing challenges faced by VHTs during IPT-G delivery.
Process and outcome evaluation	• In post-intervention debriefings, both caregivers and VHTs reported benefits of the intervention.
	• Caregivers formed and maintained self-help groups to sustain change.
	• Recommendations for MOH from stakeholders include dissemination of the VHTs model for IPT-G delivery and health workers to serve as facilitators; VHTs requested financial compensation to maintain services.
	• The IPT-G session notes and implementation process evaluation were used to develop a training guide for IPT-G in nodding syndrome affected areas.
	• Results from the study were used to inform a pilot of the WHO mhGAP intervention IPT component by the government.
	• Validation study of a bigger population is planned to inform scale-up

#### Recruitment of intervention participants in government health services

VHTs were able to effectively recruit an adequate number of participants. Eighty-six caregivers participated in the pre-assessment exercise, and 73 fulfilled criteria for inclusion. However, 10 caregivers who were assessed but did not meet inclusion criteria (i.e., their scores on the PHQ-9 were below the cutoff) requested to participate in the intervention, as did three other caregivers with children with NS who were not identified during the recruitment phase. Through consultation with the local leaders and health workers, we decided to include these individuals because when scaled-up into government health services, the intervention could be made available to persons requesting care with distress at subclinical levels. Similarly, once the group sessions began, two caregivers of children with nodding syndrome were allowed to join though they had initially declined participation. This is because under real-life conditions, caregivers may join at different times based on their need and timing of services. Although we originally conceptualized closed-groups, VHTs and district stakeholders stated that open-groups would enhance acceptability and utilization by community members. For the purposes of quantitative evaluation, we used an intention-to-treat approach and only included those participants who met the inclusion criteria.

#### Delivery of IPT-G within the government health system

The intervention was delivered in three stages: initiation or early phase, middle or intermediate phase, and the termination phase (Additional file 1; a sample session is available for viewing online (https://www.youtube.com/watch?v=RQ2eYn-J8w8). Supervision of the intervention by IPT-G therapists and clinical psychologist focused on adherence to IPT-G structure and addressing challenges faced by VHTs during IPT-G delivery. Modified supervision topics included follow-up of caregivers with suicidal behavior. Challenges included poor documentation, difficulties with supporting caregivers experiencing domestic violence, and motivating those caregivers with symptom resolution to stay in treatment. Peer supervision by more literate VHTs was used to support documentation for less educated VHTs. IPT-G therapists observed sample group sessions to document challenges and tailor content of refresher trainings.

#### Process and outcome evaluation

Intervention completion was documented through caregiver attendance records at each therapy session. Sixty-nine caregivers attended each of the 12 therapy sessions which translated into more than 90% of study participants. However, only 63 caregivers and children were available during the post assessment exercise (Additional file 1). Reasons for caregiver attrition included relocation to other villages because of new employment opportunities, alcoholism, loss of interest, marital breakup, and one death.

Quantitative results of the trial have been published and demonstrate the effectiveness of IPT-G in treating depression in both caregivers and their children [34]. Significant effects were also observed for psychological distress, stigma, and social support among the caregivers [34].

#### Feedback and dissemination meetings

The VHTs described positive changes among the caregivers, such as improved hygiene for the children and humane methods of care compared to pre-intervention conditions. A sense of group cohesion among caregivers was observed after the intervention. Groups unexpectedly continued to meet after completion of IPT-G for peer support and microfinance activities (*Bol Cup*). Reports of domestic violence among the caregivers had also reduced.

VHTs found the session reporting format, which required written documentation, demanding because they typically do limited documentation for government health services. VHTs reported that the experience changed their own lives with marked improvement in their personal relationships, reducing alcohol use, and self-grooming. These changes were not reported by VHTs in the control group.

Challenges faced by VHTs during implementation of IPT-G included inadequate financial compensation, managing discussions when IPT-G participants’ partners attended the sessions, and alcohol consumption which affected caregivers’ attendance and participation.

### Component 4. Maintenance and evolution

#### Scale-up

Recommendations for scale-up provided to the MOH included the above findings as well as additional post-intervention suggestions from stakeholders that primary health workers serve as IPT-G facilitators before taking on the role of supervisors. Results from the study were also used to inform a pilot of the WHO mhGAP IPT component by the MOH in northern Uganda. The final result was a training curriculum and supervision guidelines for delivery of IPT-G by VHTs.

## Discussion

To contextualize a PT in the Uganda public health system, we employed the REP components: pre-conditions, pre-implementation, implementation, and maintenance and evolution. This entailed a three-step process: review of documentation about health services for NS affected families and available evidence for PTs, qualitative preparatory activities, and process analysis of implementation.

In the pre-condition phase, we selected IPT-G, which had a local evidence base. For the pre-implementation phase, we collaborated closely with government stakeholders to adapt the training and supervision to government policies and human resources availability. Further adaptation was done for use with NS-affected populations to fit with their particular mental health needs and to harmonize with government NS treatment guidelines. During implementation, we conducted a controlled trial of IPT-G and performed a process analysis to guide further modifications. Quantitative evaluation suggested positive effects for both beneficiaries and the providers [34].

We began initial activities for the maintenance and evolution phase in collaboration with the MOH during their efforts to integrate IPT-G into WHO mhGAP nationwide scale-up.

### Relevance of contextualization to global health task-shifting

Our findings are relevant for global health task-shifting initiatives for which success and sustainability will depend highly on the degree to which EBTs are contextualized into government health systems. In Table 4, we describe how the REP framework can be used to guide contextualization for global mental health (GMH) initiatives. The degree of contextualization in GMH will vary widely. In our work with IPT-G, we were able to use materials that were previously adapted for use by non-specialists in northern Uganda. In other settings, there may be EBTs that have been adapted and successfully implemented in the region but not within the specific country or public health system; this would require significant linguistic, cultural, and contextual modification. In other instances, treatments only have an evidence base in Western cultural, high-income settings; this requires the greatest degree of investment for the contextualization process.

**Table 4:** Key steps for contextualization of evidence-based psychological interventions in low-resource health systems according to the Replicating Effective Programs (REP) framework

REP stage	Implications for contextualization of psychological treatments for low-resource health systems
1. Pre-conditions	1.1. Establish need for psychological treatments (identify and characterize target population and condition)
	1.2. Use systematic, scoping, or desk reviews to identify psychological treatments with an evidence base for target condition in similar cultural settings and health systems context (e.g., what treatments have been successfully delivered in the country or region? What treatments have been successfully delivered in similar low-resource health systems?)
	1.3. Review national and local health policies and guidelines to determine human resources in health system, levels of training of different health worker cadres, existing supervision systems, and cadres with greatest engagement with target population (e.g., What health worker cadres in the government health system can most feasibly deliver the intervention and what is their current supervision pathway?)
	1.4 Review existing training approaches for the target health worker cadre with regard to their literacy level, costs and compensation for training, types of trainers, etc. (e.g., How long could the training feasibly be? Who could deliver it? How and what materials need to be adapted?) and develop an implementation research manual
2. Pre-implementation	2.1 Conduct formative research to identify cultural beliefs and practices including coping and health seeking behavior of target beneficiaries
	2.2 Identify health system attributes and other contextual factors that may facilitate and/or obstruct access to psychological treatments in the government health system
	2.3 Partner with local administration and health system to form community advisory boards, working groups, and stakeholder groups
	2.4 Establish process for community working groups and stakeholder groups to collaboratively develop and modify implementation plan within public health system, as well as select appropriate site(s) for pilot implementation
3. Implementation	3.1 Conduct training within government health system for both supervisors and implementers of the psychological treatment
	3.2 Build technical and support supervision into government health system, while addressing potential barriers related to power differentials and hierarchies, burden of work and limited compensation, low literacy and knowledge among supervisors, and issues regarding technology use, reporting, and internal dissemination
	3.3 Use hybrid implementation trial designs to evaluate implementation and effectiveness outcomes
	3.4 Organize stakeholder feedback and dissemination meetings during and post-piloting to inform refining of intervention package and next steps
	3.5 Revise manual and materials for treatment delivery based on implementation and effectiveness outcomes
4. Maintenance and evolution	4.1 Work with government and international stakeholders (e.g., World Health Organization) to plan expansion throughout the government health system.
	4.2 Establish process for national and local quality monitoring and improvement, including ongoing evaluation of health and economic outcomes

During REP’s pre-condition phase, it is important to consider both cultural and contextual similarities. An intervention may have been adapted for the cultural context, but if it utilized high levels of expertise, training, and supervision, this would still necessitate major contextualization for government integration. In contrast, if the EBT was adapted in a different cultural setting but developed in a low-resource setting, it will require significant cultural but not contextual adaptation. Therefore, in the pre-conditions phase, we recommend exploring potential EBTs according to both cultural and contextual suitability. Given the increasing use of CHWs in delivery of PTs, there is a broadening pool of potential interventions that can be contextualized for government health system in low-resource settings [5, 8, 9, 45]. Crucially, government buy-in is needed to determine which cadre is most appropriate and sustainable.

After a PT has been selected and the health system cadre selected, the training curriculum will need to be adapted to fit with local training approaches, duration, and compensation. Adaptations to the training program for VHTs and health workers for IPT-G led to the 7-day curriculum—a compromise between the recommended 2-day training for VHTs and the 14-day duration of training for IPT-G therapists in prior NGO trials.

The supervision system also needs to be defined within the parameters of the government health system. The supervisors (primary health workers in our example) were new to the intervention as were the VHTs. Although they were trained as supervisors, they had no direct experience delivering the intervention, which greatly limited their ability to supervise VHTs. Therefore, their supervisory role may not have been to the standard desired. We attempted to address this by involving expert IPT-G therapists who had previously participated in the intervention in this region. This supervision of supervisors approach will likely be necessary in most contextualization programs until government supervisors have adequate experience. The apprenticeship model to training and supervision is a good example of how similar approaches have been used in GMH [45]. Moreover, fidelity tools that could be peer-rated for supervision and quality improvement are recommended [46].

Another contextualization issue for PT delivery in low-resource settings is the literacy level of providers. Literacy among government VHTs was lower than the standard for NGO employed IPT-G therapists. We developed a “buddy system.” VHTs with competency in English assisted “buddies” with limited literacy. This should be considered in other GMH initiatives.

In addition to training and supervision, the implementation process and fidelity are affected by recruitment, participant responsiveness, staff’s commitment to the intervention program, and staff’s ability to perform the intervention with resources at hand [47]. IPT-G had good recruitment rates indicated by the number of caregivers who were willing to participate in the intervention. High-recruitment and low-attrition rates can be useful indicators of contextual validity. The ability of a PT to benefit the non-specialist providers, which we observed, should also be a factor in task-sharing programs as it may enhance treatment allegiance.

Contextualization should also address compensation of those taking on additional tasks. The issue of compensation is an ongoing debate in global health [48–50]. Monetary support for the VHTs and additional payments for supervisors was done in our trial according to government rates to inform the adaptation and sustainability of such an initiative. However, these rates are considered low, and therefore, it was a challenge keeping the VHTs and health workers motivated. Moreover, after the intervention, VHTs would go back to receiving no compensation for these mental health activities. Our recommendation is that IPT-G should be considered as additional work for VHTs and health workers, and therefore comes with commensurate remuneration from the government. This will necessitate cost-analyses of IPT-G as an element of REP phase 4 to see if IPT-G matches the expected $1 of investment to $4 of health care and other savings [51].

Cultural adaptation and health system contextualization represent overlapping processes. The contextualization process described here has elements of cultural adaptation in real-world settings [1, 52]. Cultural adaptation aspects of IPT-G reflect what Falicov describes as “cultural attunement”; an approach that focuses on the process of intervention delivery rather than on intervention content alone and “is intended to boost engagement and retention of subcultural groups in treatment” [53].

### Strengths and limitations

Our findings are limited by the lack of fidelity measures of IPT-G and the proper training of supervisors for the intervention. In addition, we did not employ a specific contextualization documentation tool but rather relied upon extraction of themes from process notes. Future studies should consider use of a systematic contextualization documentation tool, informed by the process described here, which would also help government administrators in subsequent maintenance and evolution phases. A hybrid implementation design would be useful in this regard [54]. Also our contextualisation process was guided by the REP framework, if we had chosen a different implementation framework, that may have led to a different focus and potentially different outcomes of the contextualization process [23]. We utilized the REP process as described by Kilbourne et al.; however, there were some elements which required adaptation for implementation because of process and contextual factors. We realized that contextualization of IPT-G for NS demanded that we continuously identify and respond to barriers throughout the pre-conditions, pre-implementation and implementation phases, and not restrict it to one phase. We did not set up a CWG that could regularly meet but we set up a network of community, district, and national stakeholders with whom we regularly discussed and updated emergent issues during the implementation.

## Conclusions

Our findings highlight the major considerations and outcomes in adapting and implementing EBTs within the Uganda public health system. Using the REP framework, we demonstrate the feasibility of IPT-G delivered by LCHWs, which can inform the design of community mental health interventions. The intervention facilitated capacity building within the government health system. Modifications within the health system are also suggested to assist the integration of PTs, especially the need for training, supervision, and compensation of LCHWs. Core elements of contextualization using REP in low-resource settings should include the following: identifying the need and the evidence-based PT and developing an implementation research proposal in the pre-condition phase; setting up a stakeholder guidance group in the pre-implementation phase; formative research, training, process evaluation, support and technical supervision, and feedback and refinement in the implementation phase; and continuous engagement with stakeholder groups and re-customizing delivery of the intervention during the fourth phase. These lessons will facilitate integration of WHO mhGAP psychosocial interventions into sustainable government-delivered health services. Ultimately, REP was a beneficial framework for guiding contextualization, especially for working with government administrative systems and health officials, and program beneficiaries.

## Additional files

Additional file 1: Pre- and post-assessment form (DOCX 23 kb)

Additional file 2: Flow chart showing literature search and selection process (DOCX 33 kb)

## Abbreviations

APAI: Acholi Psychosocial Assessment Instrument
EBIs: Evidence-based interventions
EBTs: Evidence-based psychological treatments
GMH: Global mental health
IPT: Interpersonal psychotherapy
IPT-G: Group interpersonal psychotherapy
LMIC: Low- and middle-income countries
mhGAP: Mental Health Gap Action Programme
MOH: Ministry of Health
NGO: Non-governmental organization
NS: Nodding syndrome
PCO: Psychiatric Clinical Officer
PHQ: Primary health questionnaire
PMO: Principal medical officer
PSW: Psychiatry social worker
VHT: Village health team
WHO: World Health Organization
