# Supplementary material for: Contextualization of psychological treatments for government health systems in low-resource settings: group interpersonal psychotherapy for caregivers of children with nodding syndrome in Uganda
Source: Implement Sci. 2018 Jun 28;13:90. doi: 10.1186/s13012-018-0785-y (PMC6025709; doi:10.1186/s13012-018-0785-y)
Supplement: Supplementary file 1 — Pre- and post-assessment form (DOCX 23 kb) [file 13012_2018_785_MOESM1_ESM.docx]

**Additional file**

**Pre and Post assessment form**

**IPT-G CAREGIVER PRE ASSESSMENT FORM**

Caregiver’s name………………………………………………………………………… Date…………………………………

Caregiver’s Village………………………………………………………………………………….

Sex…………………………………………… Age……………………………………………

I will read you a list of symptoms which people/persons with depression have .On every symptom you will be able to tell me how you are affected within the past two weeks

Caregiver’s symptoms (Check all that apply) Frequency Duration

1. 🞏Sadness (*Cwer Cwiny)* ………………………….. …………………………………
2. 🞏Lack or loss of interest *(miti metimojamima jwiitinekiyomcwinyrwenyo e.g Pur, gwokolatino)*  …………………………….………………………………
3. 🞏Lack of or poor concentration: difficulty in making thinking or making decisions *(PeKetoCwinye)*……………………………. ……………………………………
4. 🞏Appetite problems: weight gain or loss *(Miti me cwam rwenyo*)…………………
5. 🞏Sleep problems: too much or too little *(Peko me nino)………………………………*
6. 🞏Feelings of guilt or worthlessness *(Tam ni Kony peke)*………………………………
7. 🞏Tiredness or lack of energy *(Kero me tic dokonok/rwenyo)* ………………………………………………. ………………………………………..
8. 🞏Movements feel slow *(Wotocalotipukomipeke) ………………………………………………………*
9. 🞏Suicidal behaviour *(Tam me dene rwenyokwoni)* …………………………………………………………..
10. **FEMALES: FUNCTIONALITY**

| **Activity regularly done in our communities/home** | Not affected  0 | Somehow affected  1 | Affected  2 | Very much affected  3 | **What causes this problem** |
| --- | --- | --- | --- | --- | --- |
| Keeping yourself clean |  |  |  |  |  |
| Taking care of children |  |  |  |  |  |
| Cooking food |  |  |  |  |  |
| Washing clothes and house hold utensils |  |  |  |  |  |
| Cleaning the house / compound |  |  |  |  |  |
| Digging/able to cultivate food |  |  |  |  |  |
| Participating in community development activities |  |  |  |  |  |
| Attending meetings |  |  |  |  |  |
| Supporting other community members who lose their beloved ones/participate in funeral |  |  |  |  |  |
| Others |  |  |  |  |  |

**11) MALES: FUNCTIONALITY**

| **Activity regularly done in our communities/home** | No problem  0 | Some how affected  1 | Affected  2 | Very much affected 3 | **What causes this problem** |
| --- | --- | --- | --- | --- | --- |
| Keeping yourself clean |  |  |  |  |  |
| Cultivating and keeping animals |  |  |  |  |  |
| Leadership in the home |  |  |  |  |  |
| Working hard |  |  |  |  |  |
| Looking after the family |  |  |  |  |  |
| Participating in community development activities |  |  |  |  |  |
| Participate in funeral services |  |  |  |  |  |
| Associating with other community people |  |  |  |  |  |
| Others |  |  |  |  |  |

Caregiver’s problem area (Trigger)

1.……………………………………. Grief/death of a loved one

2.……………………………………. Life change

3.……………………………………. Disagreement. What stage? …...Renegotiation……..Impasse……….Dissolution

4.……………………………………. Shyness/loneliness

Briefly explain the problem: ……………………………………………………………………………………………………………

Caregiver’s goals (List one or two, these may change during the 12 weeks.)

1) …………………………………………………………………………………………………

2)………………………………………………………………………………………………………

**Caregiver Attrition (Drop-outs) rates by village**

| **Number** | **Village** | **# enrolled** | **# (%) completed** |
| --- | --- | --- | --- |
| 1 | Alokolum | 9 | 9 (100%) |
| 2 | Wii Lugoyi | 9 | 08 (89%) |
| 3 | Langorony | 14 | 13 (93%) |
| 4 | Lamoo | 08 | 07 (88%) |
| 5 | Lapoyaokwe | 10 | 09 (90%) |
| 6 | Beyojawara | 08 | 07 (88%) |
| 7 | WangOlweny | 15 | 15 (100%) |
|  | Total | 73 | 69 (95%) |

**Delivery of IPT-G for nodding syndrome – Session by Session**

In the first group session, caregivers were encouraged by group facilitators to discuss their problem areas and possible mitigation strategies. Majority of the problem areas identified by caregivers were in the life change category including: persistent nodding syndrome in their children, domestic violence and marital conflict, living with HIV/AIDS and poverty among others.

VHTs’ session reports indicated that over 96% of caregivers had attended and were supportive to one another during sessions. Ten out of thirteen VHTs attended the support supervision meeting held at the end of the week. During the meeting, a review of the session notes indicated that 12 caregivers had active suicidal ideation and had been referred to the health facility. VHTs and the supervising health workers were guided on how to conduct immediate follow up and referral of caregivers with suicidal behaviour to the supervising health facility. Caregivers continued with discussions about their problem areas and possible solutions during the second group session. The VHTs were observed to be collaborating and consulting one another before the second group meetings. This translated into better preparedness and good management of the second treatment sessions.

In the third and fourth sessions, group facilitators noted that caregivers were more forthcoming about their problem areas.**.** Local leaders were also observed to be interested in the intervention as shown by their impromptu visits to some of the sessions. The ability of caregivers to identify triggers for their depression symptoms had noticeably improved in session four. A review of session and supervision notes indicated that more than 90% of VHTs were using appropriate IPT-G techniques in facilitating group session and addressing the interpersonal problems of caregivers.

In the fifth week of IPT-G, one of the groups, with every member’s consent, included another caregiver who had not participated in the pre-group assessment exercise. A difficult case of attempted suicide was identified during one of the group’s sessions, which necessitated the attention of local leaders and referral to the health facility. The VHTs responsible were commended for a job well done. The fifth weekly supervision meeting identified key challenges reported by the supervising health workers: some of the caregivers had difficulties with setting their weekly and long term treatment goals and, some of the VHTs had difficulties with filling the group session forms. The VHTs were supported on how to help caregivers do a problem analysis, link goals to problem areas of each caregiver**,** track progress on set goals of each caregiver**,** follow up of caregivers with severe problems and link symptom changes to events. The IPT-G therapists advised more intensive in-session supervision of VHTs by the health workers and more focus on documentation during the next supervision meetings. A recommendation to have a refresher training earlier than planned was made to address these challenges

Supervision notes from session six indicated that caregivers appreciated the approach used to resolve their problem areas. It was also noted from the supervision reports that majority of the caregivers that had not shown much improvement in their symptoms were those with domestic violence related problems. All the VHTs were showing high levels of commitment to their roles. The VHTs’ supervision focused on how to engage with caregivers that did not seem to be improving, how to explore and support caregivers with domestic violence problems, tracking progress of goals set by caregivers to manage their problems, problem analysis and challenges encountered by each caregiver in reaching target treatment goals. The VHTs were also assisted on how to correctly fill group session forms.

During session seven, caregivers were observed to be more supportive towards one another through their readiness to offer help and solutions. The VHTs’ supervision was centered on how to link attainment of goals to changes in symptoms realized by caregivers. The primary focus of the previous week’s supervision, tracking progress of goals set by caregivers to manage their problem, was also strongly emphasized. IPT-G therapists’ reports indicated that all the VHTs were conversant with basic IPT techniques and strategies used during group sessions to manage the interpersonal problems of caregivers. However, there were gaps identified in the VHT practice, especially in facilitating group sessions in which most of the caregivers have registered improvement in their symptoms. The VHTs were therefore supported on the following key areas of group facilitation: linking symptom reduction to interpersonal achievements made by each caregiver and working towards the therapy goals, analysis of different problem solving approaches within the group (the use of debate, side therapy and drama role play), and use of experiential sharing in the group to augment learning from within. During the seventh supervision meeting, it was agreed that IPT-G therapists visit some IPT-G groups in session. A refresher training for VHTs, whose emphasis would be on the middle and termination phases of the IPT-G intervention, was scheduled

All groups except one held their eighth session; the missed group session was re-scheduled. The eighth session witnessed caregivers share more positive experiences fr**o**m their participation in the group meetings. The weekly support supervision was preceded by IPT-G therapist field visits to observe groups in session. The supervision meeting with VHTs and health workers focused on how to track notable changes among caregivers and attainment of goals set by each caregiver. The VHTs were supported on the following key areas**:** exploring strategies for attainment of set goals, supporting caregivers during mourning, managing groups that had significant numbers of members who had improved and dealing with difficult caregivers during group sessions.

During sessions 9, 10 and 11, caregivers testified about the positive effects of the intervention on their lives. The ninth, tenth and eleventh weekly supervision meetings focused on reviewing the strategy VHTs should use to approach the termination phase of the intervention. All VHT session forms were reviewed for gaps in documentation, and all VHTs were supported on how to arrange their files and improve on all the necessary documentation. Specific emphasis was put on how they would manage the expectations of caregivers toward group termination.

In the twelfth session, majority of caregivers expressed positive future plans intended to change their adverse circumstances. Some of the groups had decided to continue as self-help support groups with additional objectives including: formation of saving and cooperative organisations (SACCOs), farming, problem solving, and “Merry Go Round” home visitations to offer moral or material support to the most vulnerable. Two VHTs had ‘dropped out ‘of the intervention, one because of sickness and another because of migration to another district. The remaining 12 VHTs had co-facilitated 12 IPT-G sessions as required. All the VHTs had submitted their files to the health facility for proper storage and majority of the files had complete documentation.

The twelfth weekly supervision meeting aimed at supporting the VHTs in handling post intervention responsibilities with the caregivers in the community
