# Supplementary material for: Contextualization of psychological treatments for government health systems in low-resource settings: group interpersonal psychotherapy for caregivers of children with nodding syndrome in Uganda
Source: Implement Sci. 2018 Jun 28;13:90. doi: 10.1186/s13012-018-0785-y (PMC6025709; doi:10.1186/s13012-018-0785-y)
Supplement: Supplementary file 2 — Flow chart showing literature search and selection process (DOCX 33 kb) [file 13012_2018_785_MOESM2_ESM.docx]

**FLOW CHART SHOWING LITERATURE SEARCH AND SELECTION PROCESS**

PubMed: **18**

Web of Science: **14**

PSYC INFO: **31**

Ovid Medline: **17**

No of articles excluded: **6**

Reasons: No clear description of method of delivery of psychological treatment or intervention

No of records excluded: **19**

Reasons: No reference to the delivery of a psychological treatment or intervention

Combined searches after removal of duplicates:  **34**

Number of duplicates removed **= 46**

No of studies included in data extraction and qualitative synthesis: **9**

No of full text articles assessed for eligibility: **15**

No of records screened: **34**
